# Novel application of the MSSCP method in biodiversity studies

**DOI:** 10.1002/jobm.201000117

**Published:** 2011-06-09

**Authors:** Karolina Tomczyk-Żak, Szymon Kaczanowski, Magdalena Górecka, Urszula Zieenkiewicz

**Affiliations:** 1 Institute of Biochemistry and Biophysics, Polish Academy of SciencesWarsaw, Poland

**Keywords:** MSSCP, Diversity, Biofilm, LIBSHUFF

## Abstract

Analysis of 16S rRNA sequence diversity is widely performed for characterizing the biodiversity of microbial samples. The number of determined sequences has a considerable impact on complete results. Although the cost of mass sequencing is decreasing, it is often still too high for individual projects. We applied the multi-temperature single-strand conformational polymorphism (MSSCP) method to decrease the number of analysed sequences. This was a novel application of this method. As a control, the same sample was analysed using random sequencing. In this paper, we adapted the MSSCP technique for screening of unique sequences of the 16S rRNA gene library and bacterial strains isolated from biofilms growing on the walls of an ancient gold mine in Poland and determined whether the results obtained by both methods differed and whether random sequencing could be replaced by MSSCP. Although it was biased towards the detection of rare sequences in the samples, the qualitative results of MSSCP were not different than those of random sequencing. Unambiguous discrimination of unique clones and strains creates an opportunity to effectively estimate the biodiversity of natural communities, especially in populations which are numerous but species poor. (© 2012 WILEY-VCH Verlag GmbH & Co. KGaA, Weinheim)

## Introduction

In recent decades there has been a growing interest in the analysis of natural communities. Increased insight into microbial diversity has been accompanied by progressive development of techniques employed in this research. The latest approaches used direct shotgun sequencing [1,2] and high-density oligonucleotide microarray technology [3]. Such mass methods are powerful tools which provide a lot of information but they are not very popular because of their high cost and relatively short reads. Despite the rapid development of highthroughput techniques, there are many studies based on the improvement of older methods to increase the accuracy of detection of environmental microorganisms.

The predominant molecular markers for the assessment of microbial diversity are DNA fragments of the ribosomal RNA operon [4]. The 16S rRNA gene is a good target for PCR amplification for this purpose because of its well defined regions, which enable taxonomic classification, and because of the availability of a very rich sequence database. The next step involves detecting sequence variants of PCR fragments of genetic markers either by cloning and sequencing or by electrophoretic analyses. Electrophoretic techniques include methods which enable separation of PCR products according to their length or sequence polymorphism. The most commonly used sequence polymorphism method for estimating the diversity of microbial communities is denaturing gradient gel electrophoresis (DGGE) [5]. However, this method is technically difficult and leads to underestimation of true bacterial diversity as a result of co-migration of different sequences during electrophoresis.

Single-strand conformation polymorphism (SSCP) is a widely used method for single nucleotide polymorphism (SNP) and point mutation detection [6]. Initially, this method was used to determine the diversity of natural microbial communities, for example by Lee *et al*. [7]. More recently however, several modifications of the SSCP approach, such as capillary electrophoresis (CE-SSCP) [8], multi-temperature single-strand conformation polymorphism (MSSCP) [9] or deaminated single strand conformation polymorphism (DSSCP) [10] have been proposed to improve the applicability of this type of method in different areas. For MSSCP analyses, three different temperatures are used to separate short DNA fragments, which vary in sequence but are of the same length, in non-denaturing polyacrylamide gel electrophoresis. Until now, MSSCP analyses have mostly been used for the detection of point mutations in specific genes [11,12] which are important in clinical medicine. Recently, Szewczyk *et al*. [13] used this method to detect highly conserved polyhedrin gene sequences of baculoviruses.

In the present study, we applied the MSSCP technique for the screening of unique sequences of the 16S rRNA gene library and bacterial strains isolated from a biofilm growing on the walls of an ancient gold mine in Zloty Stok in Poland. For this purpose the biofilm sample was divided into two parts, one destined for isolation of total DNA and the other for culturing of the microorganisms. The total DNA was used as a template for amplification of 16S rRNA genes. The obtained PCR products served subsequently to create the 16S rRNA gene library. The MSSCP analysis of variable V3 regions was performed both, directly on the cultures of the isolated strains and the 16S rRNA gene library to determine unique 16S rRNA sequences. Statistical verification of MSSCP analysis was done by comparison to results obtained from random sequencing of the 16S rRNA gene library.

## Materials and methods

### Biofilm sample collection

Samples of biofilm were collected aseptically into sterile tubes from the walls of the collapsed Gertruda adit, which is a part of the closed ancient gold, arsenic mine in Zloty Stok, Lower Silesia, Poland. During transport samples were kept at 4 °C.

### Isolation of bacteria

Samples of the biofilm (approximately 0.5 g each) were vortexed in 500 μ of 0.8% NaCl for 60 min at room temperature and then centrifuged for 10 min at 8000 rpm. The pellets were resuspended in 100 μ of 0.8% NaCl and spread in parallel onto agar plates with different media: LB (Difco), LB with 5 mM Na3AsO4 and Beijerinck's [15]. Cultures were kept at 14 °C, 20 °C or 37 °C, in darkness. Morphologically different isolates were transferred onto LB-agar plates to obtain pure cultures.

### DNA extraction

Total DNA was extracted essentially as described [14], but with the following modifications: combined partial samples of the biofilm (6 g) were homogenized using glass beads in the presence of the extraction buffer by shaking in a Mini Bead-Beater (Bio-Spec Products, Bartesville, U.S.A) three times for 1 min and then repetitive frozing-thawing (after the addition of 20% SDS). The DNA was purified using the NucleoSpin Tissue kit (Macherey- Nagel).

Genomic DNA of isolated biofilm strains were obtained in form of cell extracts by incubation of the bacterial colonies with lysozyme (5 mg/ml, 30 min), addition of lysis buffer and then 5 min boiling followed by centrifugation (6 min/9000 rpm).

### 16S rRNA gene amplification and cloning

16S rRNA genes were PCR amplified using PfuPlus Polymerase (EurX, Gdansk, Poland) and universal primers 27F and 1492R [16]. The templates for amplification were cell extracts of isolated biofilm strains or total biofilm DNA (3.7 ng/μ). The PCR conditions were as follows: 95 °C/5 min, 20 cycles of 95 °C/30 s, 53 °C/30 s and 72 °C 90 s, followed by 15 cycles of 95 °C/30 s, 46 °C/30 s and 72 °C/90 s.

PCR products of total biofilm DNA were cloned using Zero Blunt® TOPO® PCR cloning kit (Invitrogen) according to the manufacturer's instructions.

### Amplification of the V3 region of the 16S rRNA genes

For MSSCP analysis, V3 regions of the 16S rRNA genes were generated using primers 357F and 518R [17] and Taq polymerase (Fermentas). Both cultured biofilm strains colonies and colonies from 16S rRNA genes cloning were used as templates in colony PCR. Briefly: small part of colony was suspended into 20 μ PCR standard reaction mix containing N-N-dimethylformamide (0.8 μ) directly in PCR tube. The PCR conditions were as follows: 95 °C/5 min, 30 cycles of 95 °C/20 s, 53 °C/45 s and 72 °C/60 s.

### MSSCP

In order to conduct the MSSCP assay the DNA Pointer System (Kucharczyk TE, Warsaw, Poland) was used. PCR products of amplified V3 regions of isolated biofilm strains as well as colonies from 16S rRNA genes cloning were processed using the chemicals and procedure supplied by the manufacturer. Briefly, 2 μ of PCR products (∼400 ng/μ) were denaturated, cooled on ice and mixed with 4 μ of denaturing buffer A, heated for 10 min at 55 °C and then cooled on ice with the addition of 1 μ of denaturing buffer B. Subsequently, 6 μ of denatured sample was loaded onto a polyacrylamide gel (29:1 acrylamide:bisacrylamide). The conditions for electrophoretic separation of conformers of the V3 region were determined experimentally in respect to percentage of the gel (10%, 12%), addition of glycerol (5%, 3%, none) and different sets of temperatures. The best results were achieved on 12% polyacrylamide gel without glycerol and three temperatures of 35 °C, 25 °C and 15 °C for 40 min each. The electrophoresis was run at a constant voltage of 30 V in 0.5 × TBE. Those conditions were employed in further analyses. The gels were silver stained, scanned and visually inspected.

### Sequencing and sequence analyses

PCR products of unique 16S rRNA of clones and cultured biofilm strains were sequenced using M13F, M13R, 357F and 786F primers [18] on the ABI3730/xl Genetic Analyzer (Applied Biosystems). Sequences were assembled taking advantage of Linux programs phred/phrap/consed. Chimeric sequences were identified using the Bellerophon program (http://foo.maths.uq.edu.au/∼huber/bellerophon.pl) and then manually checked. Determined sequences were then compared with nucleotide databases using BLAST (http://blast.ncbi.nlm.nih.gov/Blast.cgi).

The LIBSHUFF version 0.96 [19] was used to evaluate the differences between random and MSSCP-selected pools of 16S rRNA sequences from gene library. The sequences were aligned using Muscle (www. ebi.ac.uk/muscle/) and the distance matrix was calculated using the DNADIST program of the PHYLIP package (v3.67). The sequences were deposited in EMBL with the accession numbers FM253565-FM253684 and FN594618- FN594697, and in GenBank with GU213114-GU213156.

## Results and discussion

To address the question of the applicability of MSSCP in biodiversity studies, we (i) compared data obtained by MSSCP with that of randomly selected 16S rRNA genes for sequencing and (ii) selected out distinctive strains from culturable microorganisms on the basis of the MSSCP analysis.

**Figure 1:**
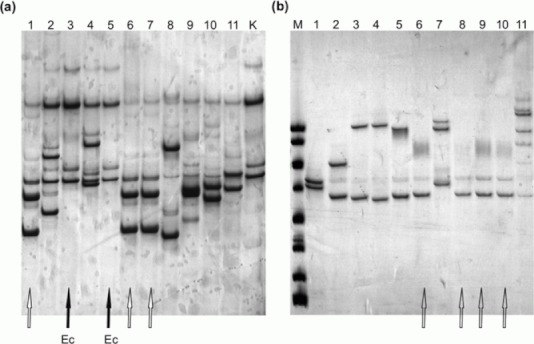
MSSCP profiles of the V3 region generated from 16S rRNA. (a) 1–11 MSSCP profiles of the V3 region generated from 16S rRNA clones. Ec – denotes profiles characteristic for *E. coli* V3 profile derived from cells with empty vector. K – MSSCP profile of V3 region generated on chromosomal DNA of *E. coli*. (b) 1–11 MSSCP profiles of V3 region generated from 16S rRNA of cultured strains isolated from the biofilm. M – 50 bp DNA ladder (Fermentas). Repeated profiles marked by arrows (identical arrows indicate the same profiles).

Firstly, data obtained from sequencing of the whole biofilm 16S rDNA was analysed statistically. Two pools of 16S rRNA sequences were generated from the gene library: randomly selected and selected by MSSCP analysis. From 4000 library clones, 10% were screened by the MSSCP method for the presence of unique V3 regions of 16S rRNA. Electrophoretic analyses showed that 51% of all profiles are unique. Some profiles were observed more than twice (Fig. 1a). Unique clones as well as representatives of the repeated clones, in total 192, were chosen for sequencing. After rejection of chimeric sequences and sequences shorter than 700 bp, 126 16S rRNA genes were obtained. In parallel, 192 randomly selected clones from the same 16S rRNA gene library were sequenced. Again, chimeric sequences and sequences shorter than 700 bp were excluded. In effect, 118 sequences were further studied.

To carry out statistical comparisons of the two approaches diversity of 16S rRNA genes was examined using LIBSHUFF [19]. The applied algorithm uses 1000 random permutations to test whether the two communities differ. The quantitative statistical analyses should consider all sequences from the pools, including repeated ones. The random pool contained redundant sequences, but the MSSCP selected pool contained only unique and had to be enlarged by adding repeated sequences. This way the redundancy in the results of V3 MSSCP analyses were taken into account. In result, 181 sequences were analysed. The probability (*P*-value) that both pools were identical was 0.001. The MSSCP analysed pool contained more sequences (181) than the pool of clones randomly sequenced (118), while the coverage value (percentage of sequences repeated more than once in a sample) was lower (45% *vs.*66%, respectively). This indicates that the diversity observed using the MSSCP approach was higher than that obtained using random sampling.

**Figure 2:**
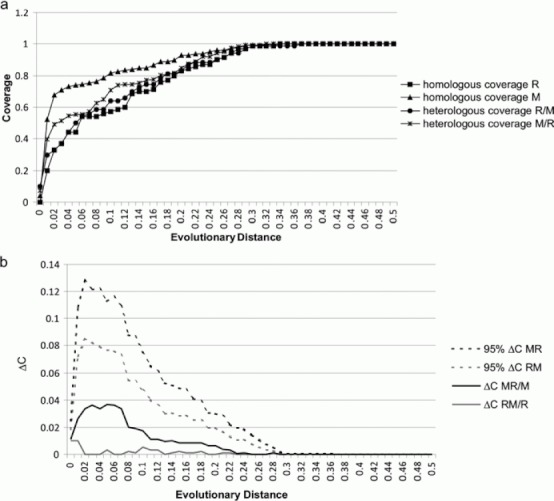
LIBSHUFF comparisons of MSSCP and random libraries. (a) Homologous and heterologous coverage curves for 16S rRNA gene sequences libraries obtained by MSSCP (M) and random (R) sequencing. (b) Squared differences between homologous and heterologous coverage (solid lines) and 95% credibility intervals obtained by permutation test (dotted lines).

Next, the samples obtained using both methods were qualitatively examined for their similarities. This time all of the repeated sequences were excluded from the analysis. Effectively, 126 unique sequences from MSSCP and 70 unique sequences from random sequencing were compared. In this case the probability that the samples obtained using both methods were identical is higher than 0.05. As shown in Fig. 2a, the coverage values (percentage of sequences identical for a given similarity cut-off point) were typical for random sampling of the identical sample. Coverage values for MSSCP (**M**) were higher than in the case of random sequencing (**R**), as expected for bigger samples. Also, coverage values for MSSCP (**M**) were higher than in case of comparison of MSSCP versus random sequencing (**M/R**). Consequently, in the case of reverse comparison (**R** *vs.***R/M**) this pattern was reversed.

The random permutation test indicated that the squared difference between homologous and heterologous coverage curves (Δ*C*) was lower than 95% credibility intervals for all evolutionary distances (Fig. 2b). This result confirms that samples were not significantly different. It is worth mentioning that the difference in °*C*-value for **M** and **R** was probably due to the difference in the size of the samples (126 *vs.*70). Both pools contained sequences assigned to the same taxonomic groups: *α-Proteobacteria*(the most populated)*, γ*-, *β*-, *ε*/*δ*-*Proteobacteria*, *Planctomycetacia*, *Verrucomicrobiae*, *Chloroflexi*and *Bacteroidetes*.

We also checked whether the MSSCP approach could be used for genotyping of bacteria strains obtained from environmental samples. Parts of the biofilm samples destined for individual culture yielded a total of 212 morphologically diverse pure bacterial cultures. The individual strains isolated from the biofilm samples were selected by choosing unique MSSCP profiles of their V3 fragments of 16S rRNA sequences (Fig. 1b). As a result, 70 profiles were defined as unique. Cells of corresponding strains were used to amplify the 16S rRNA gene sequences and the subsequent PCR products were then sequenced. The group of biofilm strains that were unambiguously identified contained species of *Actinobacteria*(21), *γ*-*Proteobacteria*(13), *α*-*Proteobacteria*(2), *β*-*Proteobacteria*(1) and *Firmicutes*(4). Nineteen strains were assigned to genera: *Micrococcus*, *Stenotrophomonas*, *Staphylococcus*, *Pseudomonas*and *Serratia*and four strains were ascribed as sequences not yet characterized.

The MSSCP method of genetic profiling enables quick, simple and cheap estimations of environmental diversity by separating short DNA fragments according to nucleotide sequences and, consequently, preselects DNA sequences as unique prior to sequencing. Among 400 analysed clones, 192 unique 16S rRNA genes were sequenced. Only one of them was repeated which confirmed the effectiveness of the applied method.

In comparison with the most commonly used electrophoretic method – DGGE – standardization of conditions in the case of MSSCP was both easier and faster. Although, for MSSCP analyzes, in contrast to the DGGE, it is necessary to obtain each sample separately, the method enabled simple differentiation of MSSCP profiles due to the presence of several bands, each of which corresponded to different conformers of the analysed DNA. Using standardized for variable V3 fragment MSSCP, a relatively large number of species in a microbial environmental sample can be detected in a cost-effective way. Due to the selection of gels with the highest sequence diversity, the applied MSSCP approach was significantly biased toward detection of rare species. However, this bias did not cause the results of MSSCP to be qualitatively different from results obtained by random sampling used as a gold standard. This means that there was no bias of detected species toward a particular systematic group.

Since the presented analysis concerned unusually rich and complex microbial community of rock biofilm, the percentage of unique sequences among the 16S rRNA clones was relatively high (∼50%). We postulate that unambiguous discrimination of unique clones and strains in populations which are numerous but species poor should be much more effective.
